# Bibliometric analysis in Scopus of scientific production on the relationship between periodontitis and gastrointestinal cancer (2014 - 2023)

**DOI:** 10.4317/medoral.26969

**Published:** 2025-01-26

**Authors:** Angel Steven Asmat-Abanto, Alfredo Portocarrero-Reyes, Rosita Elena Espejo-Carrera, Carlos Alberto Minchón-Medina, Daphne Jannet Timaná-Palacios

**Affiliations:** 1 Orcid: 0000-0001-5726-6692. Doctor in Stomatology. Specialist in Periodontics. Professor of Human Medicine Study Program - Universidad Privada Antenor Orrego, Trujillo, Peru. Professor of Stomatology Study Program - Universidad Privada Antenor Orrego, Trujillo, Peru; 2 Orcid: 0000-0001-6574-4922. Doctor in Dental Sciences. Specialist in Periodontics. Professor of Stomatology Study Program - Universidad Privada Antenor Orrego, Trujillo, Peru; 3 Orcid: 0000-0002-0247-6729. Master of Science in Clinical Research. Professor of Stomatology Study Program - Universidad Privada Antenor Orrego, Trujillo, Peru; 4 Orcid: 0000-0002-2441-5302. Professor of Faculty of Physical Sciences and Mathematics, Department of Statistics, Universidad Nacional de Trujillo, Trujillo, Peru.; 5 Orcid: 0000-0001-9433-9723. Professor of Faculty of Physical Sciences and Mathematics, Department of Statistics, Universidad Nacional de Trujillo, Trujillo, Peru

## Abstract

**Background:**

Recent studies have suggested that some opportunistic periodontal pathobionts have oncogenic properties. However, few bibliometric studies investigate the relationship between periodontitis and gastrointestinal cancer. This bibliometric study aimed to analyze these epidemiological studies conducted between 2014 and 2023 to guide future research.

**Material and Methods:**

In March 2024, the Scopus database was explored. The articles selected were subjected to a bibliometric analysis of study designs, trends in annual scientific production, and networks of collaboration among countries. Furthermore, the most outstanding countries, academic institutions, authors and journals with the most significant number of publications and the top most cited articles were analyzed. For this purpose, Microsoft Excel, SPSS and VOSviewer 1.6.20 were used.

**Results:**

A total of 123 documents were included for analysis. China contributed the most significant number of publications (33 articles) and the United States had the most significant number of citations (2709). Weimin Ye (h-index:81) and Dominique Michaud (h-index:73) were the most prolific authors (5 articles); Dominique Michaud also had the most citations (470). International Journal of Cancer was the journal with the highest number of articles published (6), in addition to being the publication that had the most citations of these articles (409). Tufts University in the United States had the highest number of citations.

**Conclusions:**

Between 2014 and 2023, 123 articles on the relationship between periodontitis and gastrointestinal cancer were published. The largest scientific production was found in China, and the most cited articles were those from the United States. Likewise, the research design most commonly used was the cross-sectional type.

** Key words:**Periodontitis, periodontal diseases, neoplasms, cancer, gastrointestinal neoplasms, gastrointestinal cancer.

## Introduction

Periodontitis is a multifactorial chronic inflammatory disease associated with dysbiotic biofilm, characterized by progressive destruction of the structures that support the teeth. Globally, it is one of the most prevalent non-communicable chronic diseases, and, in its severe form, affects between 14% and 20% of adults (1,2). This disease is a significant public health problem that negatively affects chewing, alters the appearance of those affected, and undermines their confidence and, consequently, their quality of life. Similarly, periodontal diseases can expose people to enormous socioeconomic burdens (3).

Approximately one-third of recent studies in the field of periodontics have examined the relationship between periodontitis and systemic conditions, including diabetes, cardiovascular and respiratory diseases, Alzheimer's disease, and certain types of cancer. It has been hypothesized that periodontal microbial aggression and the associated proinflammatory cascade contribute to the pathogenesis of these systemic problems (4,5).

Cancer is a disease of uncontrolled proliferation by transformed cells that are subject to evolution by natural selection, with genetic and epigenetic changes that lead to a lethal phenotype (6).

In 2022, there were nearly 9.7 million deaths from cancer recorded worldwide, of which over 3.5 million involved organs of the digestive system. Colorectal cancer ranks second in incidence and third in mortality among all types of cancer (7).

Recent studies have integrated the microbiome from the mouth to the rectum, indicating that some opportunistic oral pathobionts with oncogenic properties may emerge. The translocation of these periodontal pathobionts and the hematogenous spillage of subgingival proinflammatory mediators may potentiate the carcinogenic agents (8). In this sense, chronic gastrointestinal inflammation promotes carcinogenesis in many neoplastic diseases (9).

Considering that research on periodontitis and gastrointestinal cancer is increasing, the present bibliometric analysis was carried out to establish the distribution of articles according to study design and primary location of cancer, the trend of scientific production over the years, countries and institutions with the highest number of publications and citations, networks of collaboration among countries, authors and journals with the highest levels of scientific production and articles most cited.

## Material and Methods

A retrospective, cross-sectional and descriptive study with a bibliometric approach was conducted. In this sense, a systematic search in the Scopus database was carried out, using the variants of the keywords extracted from the Medical Subject Heading thesaurus (MESH). The formula for the search was as follows: (periodonti* OR pericementi* OR periodontal OR periapical) AND ((“digestive system neoplasms” OR “gastrointestinal neoplasms”) OR (“stomach neoplasm*” OR “gastric neoplasm*” OR “cancer of stomach” OR “stomach cancer*” OR “gastric cancer*” OR “cancer of the stomach”) OR (“colorectal neoplasm*” OR “colorectal tumor*” OR “colorectal cancer*” OR “colorectal carcinoma*” OR “adenomatous polyposis coli” OR “Gardner syndrome” OR “colonic neoplasms” OR “colitis-associated neoplasms” OR “sigmoid neoplasms” OR “rectal neoplasms” OR “anus neoplasms”) OR (“gallbladder neoplasm*” OR “cancer of gallbladder” OR “gallbladder cancer*” OR “gall bladder cancer*” OR “cancer of the gallbladder” OR “biliary tract neoplasms”) OR (“liver neoplasm*” OR “hepatic neoplasm*” OR “cancer of liver” OR “hepatocellular cancer*” OR “hepatic cancer*” OR “liver cancer*” OR “cancer of the liver” OR “adenoma, liver cell” OR “carcinoma, hepatocellular”) OR (“pancreatic neoplasm” OR “pancreatic carcinoma*” OR “pancreatic acinar carcinoma*” OR “cancer of pancreas” OR “pancreas cancer*” OR “pancreatic cancer*” OR “cancer of the pancreas” OR “adenoma, islet cell” OR insulinoma OR “carcinoma, islet cell” OR gastrinoma OR glucagonoma OR somatostatinoma OR vipoma OR “carcinoma, pancreatic ductal” OR “pancreatic intraductal neoplasms”) OR (“esophageal neoplasm” OR “cancer of esophagus” OR “cancer of the esophagus” OR “esophagus cancer” OR “esophageal squamous cell carcinoma”)). The following filters were used: range limited between 2014 and 2023, type of document type limited to “Article,” stage of publication limited to “Final,” and type of source limited to “Journal.”

- Selection criteria

Inclusion criteria

Research articles that evaluated the relationship between periodontitis and gastrointestinal cancer published between 2014 and 2023 in the Scopus database.

Systematic reviews, clinical trials, cohort, case-control and cross-sectional studies.

Exclusion criteria

Articles with incomplete information.

Narrative review articles, editorials, letters to the editor, commentaries, case reports or case series, *in vitro* studies, conference papers, book chapters, notes, letters and errata.

- Bibliometric indicators and data analysis

Scientific production per year, citations, total strength of co-authorship links, Field-Weighted Citation Impact (FWCI) and the h-index of authors reported in Scopus were included. Tables and graphs were prepared on study designs, trends in annual scientific production, and networks of collaboration among countries. Furthermore, the top countries, academic institutions (according to the first author's affiliation), authors, and journals with the largest number of publications and the top most cited articles were presented. Microsoft Excel, SPSS and VOSviewer 1.6.20 were used for this analysis.

## Results

Using the search strategy, dated March 22, 2024, a total of 807 metadata in .csv format of studies evaluating the relationship between periodontitis and gastrointestinal cancer were downloaded from Scopus. After taking into account the selection criteria, 123 articles were finally analyzed. These articles were selected and classified according to primary cancer location and research design. In some cases, the studies considered more than one cancer location, so these are not mutually exclusive, as shown in Table 1. A higher frequency of studies that related periodontitis and oral cancer [39%] was found, while the relationship least studied was that between periodontitis and gallbladder cancer [0.8%]. Cross-sectional and cohort designs were the most common, while interventional trials and umbrella reviews were the least studied.

**Figure 1:**
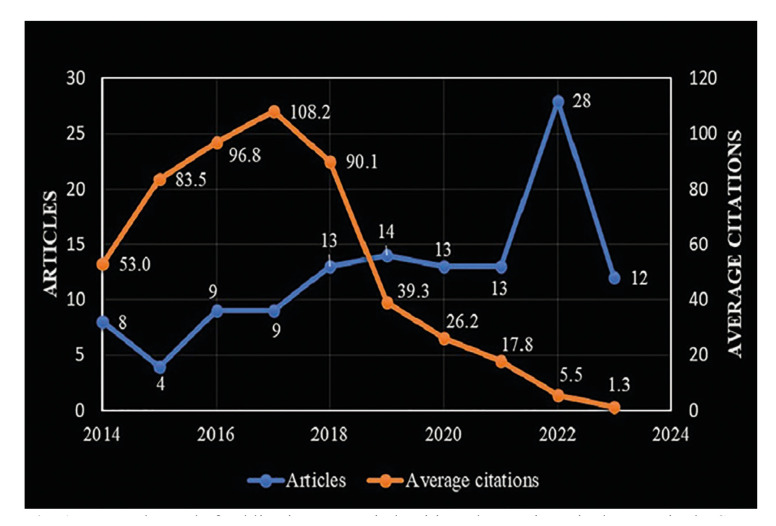
Research trend of publications on periodontitis and gastrointestinal cancer in the Scopus Database (2014 - 2023).

As shown in Fig. 1, concerning the evolution of scientific production, there was a slight trend towards an increase in publications from 2014 to 2019, with this remaining unchanged until 2021 and then showing significant growth in 2022, with double the number of cases when compared with the previous three years. However, in 2023, this was reduced to less than half compared with the year earlier. Concerning the mean number of citations per article, these increased from 2014 [53] to 2017 [108.2], then decreased rapidly until 2023 [12].

In total, 35 countries with publications on the subject studied were found. In this sense, Table 2 presents the top countries, with a limit of a minimum of 4 articles. China [33] and the United States [29] were the countries with the highest level of scientific production; the latter was also the country that had the highest number of citations in these articles [2709 citations and an average of 93.4 citations]. Furthermore, Switzerland and China were the most collaborative countries, showing a total strength of 15 and 14, respectively.

**Figure 2:**
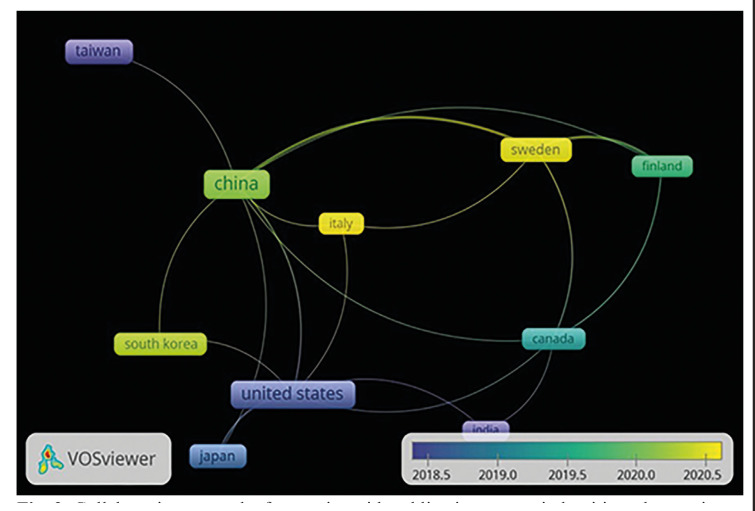
Collaboration network of countries with publications on periodontitis and gastrointestinal cancer in the Scopus Database (2014 - 2023).

Fig. 2 shows the networks of collaboration among the countries grouped into five communities: The United States, Japan, and South Korea were in community 1; Sweden and Finland in 2; Canada and India in 3; China and Taiwan in 4; and Italy in 5. The countries that most recently addressed the issue were Italy and Sweden.

**Figure 3:**
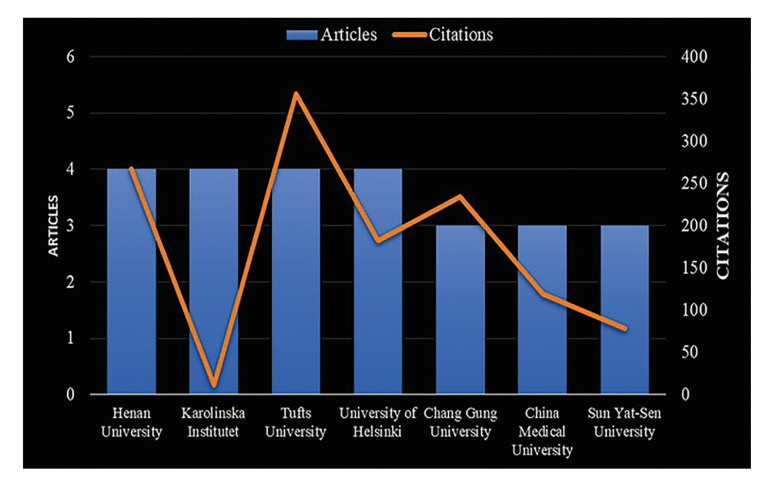
Top institutions with the highest total citations on periodontitis and gastrointestinal cancer in the Scopus Database (2014 - 2023).

The studies analyzed involved researchers from 629 organizations, some units within a single institution. Fig. 3 shows the top academic institutions with a minimum of 3 articles published, based on the first author's affiliation. The institution with the highest number of citations in published articles was Tufts University, while the institution with the lowest was Karolinska Institute.

In total, 959 authors were found, with an average of 7.8 authors/article. As shown in Table 3, Weimin Ye and Dominique Michaud were the authors with the most articles [5 each]. Christian Abnet had the highest number of citations [557], with an average of 185.7 citations/article, while Li Jin had the highest h-index [93]. Moreover, it could be seen that Weimin Ye, Xingdong Chen, Li Jin, and Ming Lu had a higher level of participation in collaborative networks [total strength = 9].

**Figure 4:**
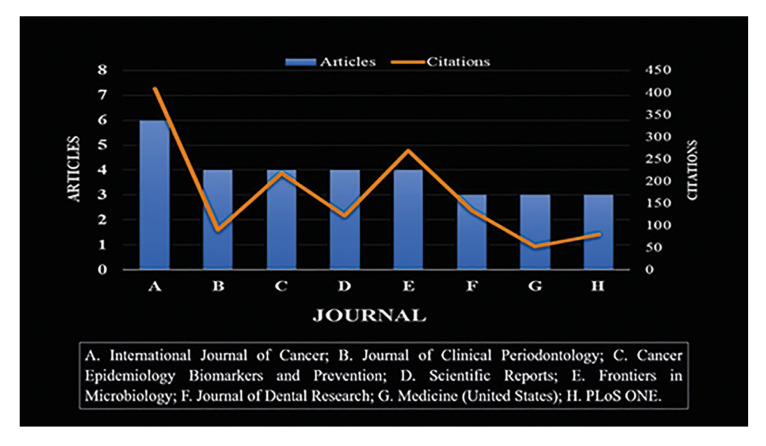
The most productive scientific journals on periodontitis and gastrointestinal cancer in the Scopus Database (2014 - 2023).

Between 2014 and 2023, 87 journals published articles on the relationship between periodontitis and gastrointestinal cancer, as reported in Fig. 4, which had a minimum of 4 articles. The International Journal of Cancer was the journal with the highest number of articles published [6] and the one that received the most citations [409]. The dental journals that published the most articles were the Journal of Clinical Periodontology [4] and Journal of Dental Research [3], with 90 and 133 citations, respectively.

Eleven of the articles analyzed had more than 100 citations, as shown in Table 4. The most frequently cited article was a case-control study on the human oral microbiome and risk of pancreatic cancer, published in The BMJ journal Gut, which had 506 citations. In general, the articles most frequently cited referred to the relationship of the oral microbiome, which generally includes the periodontopathogenic type, with pancreatic [4], esophageal [3], oral [3], and gastrointestinal cancer. Likewise, cross-sectional types were the most commonly used designs [5].

## Discussion

The relationship between periodontitis and cancer has been widely recognized, with the finding that the presence of some periodontal pathogens such as *Porphyromonas *gingivalis**, *Prevotella intermedia*, and *Tannerella forsythia* increase the risk of gastrointestinal cancer, especially in the oral cavity (8).

The bibliometric indicators aim to optimize the allocations to research and make funding more efficient. This type of analysis provides an overview of the literature in a specific field and is often used to decide the research direction (10).

In the present bibliometric analysis, cross-sectional, case-control, and cohort studies were the most frequent types. These designs have significant value for generating hypotheses and assessing evidence, considering clinical trials would be unethical or unfeasible in these studies (11). In addition, the relationship most frequently studied was periodontitis and oral cancer, possibly because the bacterial dysbiosis of the periodontal pockets and saliva would first affect the oral cavity and then translocate to other body regions (8).

Analysis of the number of publications helps identify the interest of researchers in different research fields. In this study, it was observed that between 2014 and 2019, the number of publications on the subject studied increased, with a higher rise in 2022 and a significant reduction in 2023. This finding was consistent with the situation reported in the bibliometric analysis of Gu *et al*. (12) and Hu *et al*., (13) about the oral microbiome and risk of cancer. Concerning the decline in 2023, this may have been because this type of work begins by collecting population data. Then, as more consistent knowledge is acquired, the need arises to conduct more specific research. This, in turn, requires an increase in investment, specialized equipment, and the need to conduct studies with a higher level of evidence, such as clinical trials, that would not be feasible due to the topic's characteristics. For this same reason, the number of citations could be affected.

China and the United States were the countries with the highest levels of scientific production and numbers of citations. These countries also appeared to lead the rankings in similar bibliometric studies, such as those by Hu *et al*. (13) and Li *et al*. (14). Sweden, however, with an even lower number of publications, was ranked as the country with the greatest collaborative strength. This may be because European countries tend to promote international and multi-institutional research, mainly among members of their community (14). Considering that racial/ethnic identity and genetic characteristics may be factors involved in the relationship between periodontitis and cancer concerning mortality rates and response to treatment, the findings of this study are a valuable resource for developing future dental research strategies (15).

Concerning collaborative networks, although oncological research is a global priority, it is highly biased towards high-income countries, highlighting their robust research, financing, and international cooperation systems (16,17). The United States and Japan are among the countries that have received the most public and philanthropic investment for cancer research (18). At the same time, European countries such as Italy and Sweden also invest resources, promoting various initiatives, programs, and international collaborations (19-22). The European countries are at the forefront of cancer research, contributing significantly to advances in its prevention, diagnosis and treatment (22). These collaborative efforts have the potential to foster exchange of knowledge and promote the advancement of research (23).

Tufts University and Henan University are ranked top with the highest citations. This would reaffirm the continued scientific activity of the United States and the emerging position of China. Identifying the leading institutions in this field of research allows for collaborative strategies to be developed to promote the advancement of research in this field and monitor the information generated by these institutions (13,14).

Ye and Michaud were the authors with the highest number of publications, while Abnet and Michaud were the most cited. Likewise, the author with the longest research career on the subject was Jin, with an h-index of 93. Ye and Jin are from Fudan University, while Michaud and Abnet are from Tufts University and the National Cancer Institute. This is consistent with the finding that the United States and China were the countries leading scientific production on this topic. This information allows us to identify the most outstanding researchers in the area, manage collaborations and consultations, and/or invite them to scientific competitions.

The International Journal of Cancer had the highest number of published articles and citations. This journal is a crucial source of information on advances and discoveries in this field and has a high impact factor (23).

Publications in high-impact journals allow for broader dissemination and advancement of scientific knowledge due to their high citation rate, rigor in peer review, and influence on the academic and professional community (24). Since the Journal of Clinical Periodontology and the Journal of Dental Research are high-impact journals in dentistry, they allow for significant dissemination of this topic among these professionals.

The article most cited refers to the relationship between the oral microbiome and pancreatic cancer, a particularly aggressive neoplasia with a high mortality rate, which indicates the interest of researchers. This article presented a case-control design helpful in investigating rare diseases since it facilitates the study of possible risk factors. Moreover, because it does not require monitoring many subjects for long periods, it allows these studies to be relatively fast and inexpensive (23,25); however, it provided information of moderate quality (23,24). Among the 11 investigations most frequently cited, the majority were cross-sectional studies, valuable designs in epidemiological research because they are studies that are quick and inexpensive to conduct and provide information on the prevalence and relationships between variables at a specific time (23).

The findings of the present bibliometric analysis provide the scientific community with an overview of the research landscape on periodontitis and gastrointestinal cancer, highlighting key research directions, trends, and potential future areas of exploration (22).

## Figures and Tables

**Table 1:** Research designs and studies according to the primary location of gastrointestinal cancer in the Scopus Database (2014 - 2023).

Primary location of cancer ^#^	Study design (%)						Total
	Cross-sectional	Case-control	Cohort	Interventional trial	Systematic review	Umbrella review	
Oral	21.1	8.9	4.9	0.0	4.1	0.0	39.0
Colorectal	3.3	8.1	6.5	0.8	1.6	0.0	20.3
Esophagus	4.1	4.9	6.5	0.0	0.8	0.0	16.3
Stomach	3.3	2.4	8.9	0.0	0.8	0.0	15.4
Pancreas	4.9	1.6	5.7	0.0	3.3	0.0	15.4
Pharynx	0.8	4.1	1.6	0.0	0.0	0.0	6.5
Liver	0.8	0.0	1.6	0.0	0.8	0.0	3.3
Digestive system	1.6	0.0	0.0	0.0	0.8	0.8	3.3
Intestine	0.0	0.0	2.4	0.0	0.0	0.0	2.4
Gallbladder	0.0	0.0	0.8	0.0	0.0	0.0	0.8

# Not exclusive in the studies.

**Table 2:** Top countries on the number of published papers on periodontitis and gastrointestinal cancer in the Scopus Database (2014 - 2023).

Country^#^	Articles	Citations	Average citations	Total link strength
China	33	1018	30.8	14
United States	29	2709	93.4	9
Taiwan	16	643	40.2	1
Japan	12	880	73.3	4
Sweden	12	327	27.3	15
South Korea	8	170	21.3	2
Canada	7	177	25.3	7
Finland	6	230	38.3	9
Italy	5	76	15.2	3
India	4	79	19.8	2

^# ^Countries with at least four publications.

**Table 3:** Most productive authors on periodontitis and gastrointestinal cancer in the Scopus Database (2014 - 2023).

Author	Articles	Citations	Average citations	Total link strength	h-index
Ye, Weimin	5	93	18.6	9	81
Michaud, Dominique S.	5	470	94.0	0	73
Sorsa, Timo	4	182	45.5	8	81
Chen, Xingdong	3	83	27.7	9	28
Jin, Li	3	83	27.7	9	93
Lu, Ming	3	83	27.7	9	27
Haglund, Caj	3	101	33.7	8	59
Hagström, Jaana	3	101	33.7	8	32
Yucel-Lindberg, Tülay	3	99	33.0	6	30
Abnet, Christian C.	3	557	185.7	0	47
Pan, Yaping	3	119	39.7	0	29

**Table 4:** Top-cited articles on periodontitis and gastrointestinal cancer in the Scopus Database (2014 - 2023).

Title^#^	Authors	Design	Citations	FWCI
Human oral microbiome and prospective risk for pancreatic cancer: A population-based nested case-control study	Fan X.; Alekseyenko A.V.; Wu J.; Peters B.A.; Jacobs E.J.; Gapstur S.M.; Purdue M.P.; Abnet C.C.; Stolzenberg-Solomon R.; Miller G.; Ravel J.; Hayes R.B.; Ahn J. (2018)	Case-control	506	22.98
Human microbiome Fusobacterium nucleatum in esophageal cancer tissue is associated with prognosis	Yamamura K.; Baba Y.; Nakagawa S.; Mima K.; Miyake K.; Nakamura K.; Sawayama H.; Kinoshita K.; Ishimoto T.; Iwatsuki M.; Sakamoto Y.; Yamashita Y.; Yoshida N.; Watanabe M.; Baba H. (2016)	Cohort	290	7.71
Oral microbiome composition reflects prospective risk for esophageal cancers	Peters B.A.; Wu J.; Pei Z.; Yang L.; Purdue M.P.; Freedman N.D.; Jacobs E.J.; Gapstur S.M.; Hayes R.B.; Ahn J. (2017)	Case-control	257	6.87
Association of Fusobacterium species in pancreatic cancer tissues with molecular features and prognosis	Mitsuhashi K.; Nosho K.; Sukawa Y.; Matsunaga Y.; Ito M.; Kurihara H.; Kanno S.; Igarashi H.; Naito T.; Adachi Y.; Tachibana M.; Tanuma T.; Maguchi H.; Shinohara T.; Hasegawa T.; Imamura M.; Kimura Y.; Hirata K.; Maruyama R.; Suzuki H.; Imai K.; Yamamoto H.; Shinomura Y. (2015)	Cross-sectional	250	3.74
Periodontal disease, tooth loss, and cancer risk	Michaud D.S.; Fu Z.; Shi J.; Chung M. (2017)	Systematic review	249	5.49
Presence of Porphyromonas gingivalis in esophagus and its association with the clinicopathological characteristics and survival in patients with esophageal cancer	Gao S.; Li S.; Ma Z.; Liang S.; Shan T.; Zhang M.; Zhu X.; Zhang P.; Liu G.; Zhou F.; Yuan X.; Jia R.; Potempa J.; Scott D.A.; Lamont R.J.; Wang H.; Feng X. (2016)	Cross-sectional	214	5.09
Oral microbiota community dynamics associated with oral squamous cell carcinoma staging	Yang C.-Y.; Yeh Y.-M.; Yu H.-Y.; Chin C.-Y.; Hsu C.-W.; Liu H.; Huang P.-J.; Hu S.-N.; Liao C.-T.; Chang K.-P.; Chang Y.-L. (2018)	Case-control	208	7.78
Porphyromonas gingivalis promotes invasion of oral squamous cell carcinoma through induction of proMMP9 and its activation	Inaba H.; Sugita H.; Kuboniwa M.; Iwai S.; Hamada M.; Noda T.; Morisaki I.; Lamont R.J.; Amano A. (2014)	Cross-sectional	163	2.80
The microbiomes of pancreatic and duodenum tissue overlap and are highly subject specific but differ between pancreatic cancer and noncancer subjects	Del Castillo E.; Meier R.; Chung M.; Koestler D.C.; Chen T.; Paster B.J.; Charpentier K.P.; Kelsey K.T.; Izard J.; Michaud D.S. (2019)	Cross-sectional	119	8.96
Periodontal disease, edentulism, and pancreatic cancer: a meta-analysis	Maisonneuve P.; Amar S.; Lowenfels A.B. (2017)	Systematic review	118	3.52
Periodontal pathogens are a risk factor of oral cavity squamous cell carcinoma, independent of tobacco and alcohol and human papillomavirus	Ganly I.; Yang L.; Giese R.A.; Hao Y.; Nossa C.W.; Morris L.G.T.; Rosenthal M.; Migliacci J.; Kelly D.; Tseng W.; Hu J.; Li H.; Brown S.; Pei Z. (2019)	Cross-sectional	102	4.02
